# Implementing active care through (cultural) activities of daily living: A person‐centred approach to achieve flourishing

**DOI:** 10.1002/nop2.242

**Published:** 2019-02-14

**Authors:** Janet Ververda, Solveig Hauge

**Affiliations:** ^1^ Faculty of Health and Social Sciences, Department of Nursing and Health Sciences University of South‐Eastern Norway (USN) Porsgrunn Norway

**Keywords:** active ageing, activities, education programme, flourishing, healthy ageing, narrative, Norway, nurses, person‐centred, workplace

## Abstract

**Aim:**

To investigate how participants in the Active Care education programme experienced using their obtained knowledge in practice.

**Design:**

A narrative inquiry as described by Clandinin and Connelly.

**Method:**

A thematic analysis of 20 narratives written by participants in the education programme from 2015–2017.

**Results:**

Three themes appeared to be dominant: the consequences of activity, relationships and positive emotions. These elements reflect the notion of flourishing.

## 1 INTRODUCTION

Health and activity level in older age are seen as a summation of the circumstances and actions of an individual during their whole life span. This life course approach presents opportunities, as people are able to influence how they age by adopting healthier lifestyles and by adapting to age‐associated changes. This view is known as active ageing (Edwards, 2002; Stein & Moritz, 1999). Since this view centres on the individual person, a reorientation to an individualized or person‐centred approach is needed (McCormack & McChance, 2017; WHO, 2015). Health and social services need to be aware of this reorientation and to obtain tools on how to implement a person‐centred life course approach. One of these tools is an education programme. Although several programmes exist with the objective to promote active ageing, most are aimed at specific professions, at specific diagnoses or at specific contexts (van Eerd et al., 2016; Fernandez‐Ballesteros, 2008). Additionally, education programmes are difficult to evaluate in healthcare fields because of their complexity (Haji, Morin, & Parker, 2013). This paper aims to investigate how participants experienced using the knowledge they gained from the Active Care education programme in practice. This insight may contribute to further understanding about how to implement the notion of active ageing through (cultural) activities of daily living.

## 2 BACKGROUND

In Norway, the concept of active ageing is central in several reports to the Norwegian parliament and was described as one of the five areas of strategic priority in the future (Meld.St.nr. (2011–2012) (2012); Stortingsmelding nr. 25 (2002–2003) & Stortingsmelding nr. 48 (2005–2006); Rundskriv I‐5/, 2007). Active ageing is defined as “a holistic approach for care” with emphasis on culture, activities and well‐being as central and basic elements (Stortingsmelding nr. 25 (2002–2003). This holistic approach consists of Activities of Daily Living (ADL), which includes varied and adapted activities for different users. Within this holistic approach, befrienders could play a role, as can the establishment and use of cultural‐ and daycentres. Food and meals are seen as not only a necessary good but also as an opportunity for social meeting. The arts and other cultural and sporting events can be used as therapeutic interventions or as ADL, as well as physical activity or as an experience itself (Lorentzen, 2017).

In 2012, the University of South‐Eastern Norway (USN) was commissioned by the Department of Health to assess professional development in the area of active ageing. The assessment showed that many communities offer (cultural) activities in different forms, to many different individuals and groups. However, systematic implementation of active ageing approaches was lacking in most places (Disch & Lorentzen, 2012). This may indicate that the concept of active ageing as a holistic and person‐centred approach for care is not understood and/or prioritized properly.

Based on the USN assessment, it was concluded that a national education programme was needed if the notion of active ageing was to become part of future care in Norway. The Department of Health asked the USN to develop that programme. The programme called Active Care (Aktiv Omsorg) started in 2015 and is described in detail by Lorentzen (2017).

## 3 THE EDUCATION PROGRAMME

The aim of the Active Care education programme is to educate carers on how they can offer their users the opportunity to promote, restore and maintain health through (cultural) activities. Active Care was aimed at healthcare workers and unit managers, in addition to other providers involved in other care services. Participants for the programme were recruited through advertising and by emails sent to all municipalities in Norway. The municipalities were asked to spread the invitations throughout their care‐ and cultural sectors as well as their regional and local centres to solicit volunteers. Teachers of vocational health education, national humanitarian organizations and other relevant care‐related organizations were contacted. Personal networks and key persons on the county level were also contacted. Finally, volunteer‐, sports‐ and outdoor‐related organizations were invited. These emails were followed up by phone calls from the programme's administrative staff. In total, 650 students from across Norway participated (Lorentzen, 2017).

The programme lasted eight days and was spread over four meetings. The meetings took place in a local classroom. During the meetings, the students attended lectures on Active Care related themes. Active participation and discussion in smaller groups were a means to relate these themes to their own practice. Students gained further understanding by visiting places in the vicinity where active ageing concepts were implemented. This allowed students to integrate theory into practice and to obtain better knowledge about local activities with the intention of using these activities in their work. In cases where a visit was not possible, representatives from those local activities gave classroom presentations.

The first themes of the programme were “Active Ageing” and “Action Learning.” The aim of action learning is to learn from experience by understanding one's role in these experiences and to be aware of other forces influencing the experience (Tiller, 2006). This method enabled students to understand and develop their workplaces through critical and systematic reflection about daily experiences. The varied academic level of the participants was useful for learning as the different viewpoints contributed to increased reflective activity (Ghaye, 2010).

The next programme themes consisted of the (therapeutic) environment in relation to active ageing. The key ideas included working with assessment methods—and preconditions to implement these methods—related to users, as well as user participation and empowerment. In the third meeting, network and voluntary work were themes to help students identify and mobilize resources in the users’ network and the community. In the final meeting, these themes were brought together to show how active ageing concepts can be systematically implemented to achieve well‐being. All students presented their projects in class and were given feedback from fellow students and teachers.

The students were required to hand in assignments related to the themes after every meeting. Those who wished to take the exam also had to describe the overall process of their project, based on the assignments. Part of this exam included a practice narrative, which gave a detailed and nuanced picture of an experience that has been of significance, in this case for the student (Birkeland, 1998). The aim of these narratives was to contribute to reflexive activity, as well as a form of evaluation.

## 4 THE STUDY

### 4.1 Design

The research design was based on narrative inquiry. “Narrative inquiry is a way of thinking about experience as a story” (Clandinin, Cave, & Berendonk, 2016). The analysis is centred around three dimensions: temporality (past, present and future), sociality (personal, social and cultural) and spatiality (places) (Clandinin, 2006; Clandinin et al., 2016; Connelly & Clandinin, 1990). These dimensions give a better understanding and provide new knowledge as they show the persons and their interactions over time, at a particular place in relation to each other (Haydon, Browne, & Riet, 2017). As such, this design is suited to explore the experiences from the Active Care programme in the students’ daily work. The practice narratives can thus be instrumental in understanding and revealing the possible changes the programme may have brought about in practice.

### 4.2 Methods

The material consisted of narratives written by the participants as part of their final exam of the Active Care education programme. Of the 650 participants, 423 handed in a final exam paper. The papers described the systematic process from assessment to goal formulation and outcome, as well as an evaluation. The practice narrative was only part of the reflective evaluation and was used for the purpose of this paper. The narratives were a distinctive part of the individual exams and therefore convenient to access and select. They were also believed to be authentic. These authentic significant experiences could, therefore, give useable information for the purpose of this paper (Cresswell & Poth, 2018; Haydon et al., 2017).

The exams were archived, but neither names nor places of work could be seen before opening the document. In this way, narratives from all fields were equally likely to be selected (Webster & Mertova, 2007). For the purpose of this paper, 20 of these 423 exams were systematically selected by drawing every 21st exam. The material selected to be analysed consisted of 304 pages of written text, of which 19 pages were dedicated to the narratives.

The sample was seen as representative for the total population of students in the programme as most of the narratives came from community services (Lorentzen, 2017). Of the twenty narratives, 11 were related to older people. Mental health services were represented by three narratives, and there were two narratives from the area of learning disabilities. One narrative was written by someone working in the community cultural sector and one came from the Norwegian Tourist Organisation. Two were written by those working with people with a physical handicap, one of which wrote in her role as befriender. One narrative was written by someone who worked at a family counselling service in the community. All the narrators were female (Table 1).

**Table 1:** Participants in the study

Profession	Place of work	Activity	Profession	Place of work	Activity
1: Occupational therapist	Day Care centre	Reading group	11: Occupational therapist	Mental health	Training
2: Environmental worker	Day Care centre	Bike rides outside	12: Social educator	Learning disabilities	Snoezelen (a multisensory environment for people with different handicaps)
3: Nursing assistant	Institution	Wellness for hands and feet	13: Nurse	Cognitive impairment	Cycling on home trainer
4: Leader	Culture department	Line dance at day centre	14: Nursing assistant	Nursing home	Wellness
5: Nurse	Family advice centre	Outdoor activities	15: Occupational therapist	Nursing home	Gardening
6: Community worker	Mental health services	Physical activity	16: Nurse	Learning disabilities	Party
7: Nursing assistant	Mental health community	Painting	17: Nurse	Shared accommodation dementia	Better meal situations
8: Project leader	Tourist organization	To the top with refugees	18: Nursing assistant	Nursing home	Activities day
9: Befriender	Physical handicap	Animal‐assisted therapy	19: Leader	Day care centre	Birthday celebration
10: Leader	Nursing home	Boat trip	20: Nurse	Advice for older people	Social meeting place for elderly men

### 4.3 Data analysis

The narratives were descriptions of specific activities where the participant used her new knowledge about Active Care. These activities were as diverse as the narratives themselves. The narratives were analysed in three steps.

The first step was to determine what each narrator wanted to tell the reader, in line with the three dimensions of narrative enquiry: spatiality, temporality and sociality. Questions asked about the narratives included the following: what was special about this story, this event, at that particular time and place? What did it mean for the carer and the user and their relationship?

In the second step, units of texts were highlighted and labelled in categories across the narratives. The categories were labelled using the same words the participant had used in their narrative. For example, all participants used words such as smile and laughter to describe what happened during an activity. We labelled these categories smile and laughter.

After repeating this circular process several times, the categories were further established in the third step by developing subthemes and themes. For example, the categories smile and laughter were further categorized into the subtheme joy. Further, the subthemes joy and empowerment were synthesized to the theme positive emotions. Three main themes, comprising subthemes, emerged from across the narratives (Table 2). Finally, an utterance of a narrator was chosen that described the theme of the category as clearly as possible (Lieblich, Tuval‐Mashiach, & Zilber, 2011).

**Table 2:** Themes and subthemes in the study

Main theme	1. Consequence of activity	2. Relationships	3. Positive emotions
Subthemes	Spontaneous responses Doing more and more often	With users With colleagues	Joy Empowerment

### 4.4 Credibility

In narrative research, “the aim is not to discover whether the narrator's accounts are reflections of actual events, but rather to understand the meaning people attach to those events” (Polkingorne, 2007). Nevertheless, the data need to be credible to answer the research puzzle (Clandinin, 2006). Credibility refers to accessibility of the data, which is limited due to data protection laws. Every exam in this sample is the culmination of four papers and a presentation. The accuracy could be checked, if necessary by referring to previously submitted papers and presentations as well as discussions in class. Due to clearly defined assessment and exam criteria, feedback and follow‐up over time, it is felt that accuracy has been handled appropriately (Feather, Carter, Valaitis, & Kirkpatrick, 2017). With this collaboration, the meaning of the texts are represented as closely to the original situation as possible (Chase, 2013; Haydon et al., 2017).

The students were asked to describe a learning situation as authentically as possible in their narratives. The fact that a narrative was part of an exam paper may have influenced the contents of the narratives to please the reader. To overcome this challenge, it was underlined that a narrative could also describe how not to act, to show what the students had learned from that situation. In the sample, none of these narratives were found.

The narratives were written in Norwegian and were of different linguistic quality. Although great effort has been made to retain the original content of the narrative, details may have been lost in translation. This challenge was met by seeking the help of native speakers to keep the meaning of the narratives as they were intended.

In addition, there are aspects of persuasiveness and coherence related to credibility. These can be met when theoretical claims can be supported by the data and when alternative interpretations are considered (Webster & Mertova, 2007). These will be discussed further below.

### 4.5 Ethical considerations

Research Ethics Committee approval for this study was obtained from the Norwegian Centre for Research Data. Approval to use the material was obtained from the Centre of Care Research and head of the faculty at USN. Participants were informed of the objectives of the study. Written consent was obtained after all questions concerning this study, and the intended use of participants’ papers was answered. The narrators were traced back with the help from the administrative staff so that they could give their written consent to use the narratives for this paper. Confidentiality of the data was maintained.

## 5 RESULTS

The results are presented in three themes. Texts from the narratives are used to elaborate and to give nuance to the various subthemes.

### 5.1 “After a while everybody sings” (Consequence of activity)

This theme encompasses the effects that spring from an activity. As one narrator writes: “An activity is so much more than just an activity.” The narratives show that the activity itself is greatly appreciated by the users and their colleagues. However, the activity also seems to be the means to something else. For example, it creates an atmosphere that triggers spontaneous responses, from the users but also from the carers involved. As one narrator tells about their activities day for older people:

Now most people have done all the (activity)posts. It is time for food… it is lunchtime. Now there are a lot of people around the grill, users and staff and most of them have had their food. A small group starts humming a song. After a while, they sing. Then it develops into a singalong and everybody joins in.” It is described as “a good atmosphere” and “most people are happy now.

Another narrator tells about a bike trip with some of their residents. The bike trip itself is a positive experience, but more so the breaks during the trip are as follows:

After half an hour we arrive at S. where there are benches and tables. We enjoy the food and something to drink … and enjoy the view and the sun. One of our users has dementia but as soon as he saw the sea, he started telling many poignant stories about the time when he sailed as a machinist during the War.

Subthemes that refer to the consequences of an activity are “doing more” and “doing more often.” This is based on the narrators writing that they had to determine whether or not the chosen activity was doable and appropriate. The “more” is an extension of the activity itself, but is a rather spontaneous response. One narrator writes that “staff and users on the bike trip also picked flowers to take back to the residents’ rooms.” An older people user who would try the home trainer for 10 min continues to cycle without breaks for 30 min “because it is so nice” and asks “when can I cycle again?” Doing more contains the spontaneous wish to repeat the activity because of its success.

“Doing more often” means that the activity continued and/or was adjusted, based on reflexive activity. One narrator who works with people with learning disabilities tells about her experiences with organizing a party: “It was talked a lot about among the staff … Surely this is something we have to continue to do.” Another narrator at a nursing home describes the hard work to organize a boat trip. When the first trip appears to be a success “for residents, staff and befrienders” she writes that:

it inspired further work with Active Care. The ‘boat trip’ activity has been implemented several times now. Action learning has been used as a method so that we, the staff, will get better on these interventions and implementation.

Doing more” and “doing more often” are also described as having directly observable effects on physical and mental health. A narrator who writes about giving daily hand and foot massages to residents with dementia says that: “we have now made a plan so that we can continue the activity and we see that she is much more awake and her hands and feet are much softer.” Another narrator at a nursing home tells how she engaged a user in morning trim and: “We have all noticed a major change in the user. She is more with it, it is easier to work with her on her bad days and … she participates in almost all activities now.”

As one nursing assistant sums up: “It was with a good feeling I went home that day.”.

### 5.2 “You'll be alright, we can do this together” (Relationships)

This theme shows the relationship between the carer and the user but also the carer's relationship with colleagues. The importance of these relationships is shown in interactions that are built on to implement something new. These interactions are rewarding for both the user and the carer. They inspire the carers to continue to be compassionate. As one narrator tells: “He gave me a hug and said it had been really, really nice. Then it is difficult to keep your eyes dry as I only hoped it would be ok enough… so most of my colleagues say that this is something we have to continue with and it is a really good feeling, you get so happy... No one has said they do not want to participate next time.” Another narrator who works in the mental health services describes her relation with a user as follows:

She sees me, smiles and gives me a hug. Whispers that she has bought new training gear and wonders if she can use it. I answer yes, of course, you look good in it!

Another part of this theme is how the carers’ relationships with their users inspired them to continue working together as a team towards a mutual goal: “I am grateful for sharing this experience with people who have shown such joy.” Another narrator stresses the teamwork and the responses of her team:

We were all moved by it and personally I was moved and proud about how much we had achieved with our activity. It was because we worked together. We all participated and worked enthusiastically towards the same goal. We handled the challenges and supported each other all along.

### 5.3 “Oh, imagine I could experience this!! I can hardly believe it!” (Positive emotions)

Almost all narratives mentioned words such as “smile” and “laughter” several times, which may give an indication of the participants’ achievement.

Even though quite a few users had been in care for a long time and the carers thought they knew the users well, an activity brought forth a joyful side of the person that surprised the carers: “Ola bloomed that evening” or “It was so nice to see her beaming with joy.”

This theme also shows how the relationship with the carer is important and how it is built on to achieve the positive experience of that activity: “She looks at me and smiles” was a phrase used several times in the narratives.

Positive emotions can also be shared across cultures when participating in activities together. The tour leader on a trip for under‐age refugees said: “A lot of laughter, new and good stories and we are together about this. And I thought, again, that so much is different but around a bonfire we are together and share mutual experiences in a mutual atmosphere.” This type of atmosphere triggers new relations among users and between users and carers.

Furthermore, it appears to be the activities also cultivated a stronger belief in oneself. One user tells a narrator that “the dance course (with the therapists and other users) had given so much confidence to enable her to participate in an ordinary course.” Another narrative demonstrates how the activity (engagement at a gym) has major effects on a person's life:

That this person dares to show her arms with all the scars, dares to change clothes while talking to the person next to her that really is a big step in the right direction. It is a sign of coping, that gives confidence which again leads to participation in her own life!! Training is so much more than just getting sweaty.

A mental healthcare worker notices this stronger belief in oneself after engaging a user with a befriender so they can go on trips together:

I see a major change. His drinking days have reduced … it has been easier to contact staff if he needs to ask something. We see his network has increased, we see that he is making an effort to build his own network. We see that he is more conscious of what his house looks like. He says himself that his life has changed since he got to know his befriender.

## 6 DISCUSSION

The results of this study indicate that the participants of the Active Care educational programme had several positive experiences while implementing what they learned. The participants experienced that the activity they tried led to more and new activities, to new relationships and to positive emotions. Our findings indicate that the education programme brought about positive changes not only for the participants, but also for their users and colleagues. However, the results appear to be something more than only increasing good relationships and positive emotions. The phenomenon of “flourishing” sprang to our minds.

Flourishing has been described as consisting of positive emotion, engagement, relationships, meaning and accomplishment (PERMA; Dewing & McCormack, 2017). However, several definitions exist (Hone, Jarden, Schofield, & Duncan, 2014). Flourishing is a state of being and is an essential part of being human. Flourishing happens when people connect through meaningful and intentional practices, in healthful reciprocal relationships (Perkins, Brady, Engelmann, Larson, & Shultz, 2010; Summer, 2013). Flourishing can be built within individuals, teams and even whole organizations (Dewing & McCormack, 2017).

Our findings indicate that the effect of the activity our participants describe in their narratives strongly coincides with the definition of flourishing. According to the participants’ experiences, both the carer and the users experienced positive emotions and relationships. These experiences were facilitated by meaningful and intentional activities. One can question if this supported a humanization which contributes to people's sense of meaning, strength and belonging and is empowering (Jacobs, van derZijp, vanLieshout, & van Dulmen, 2017). Overall, we conclude that our findings indicate that the Active Care education programme has contributed to flourishing.

Based on this interpretation, one could suggest that the concept of flourishing ought to be given a central place in health care (Edgar & Pattison, 2016; Venkatapuram, Ehni, & Saxena, 2017). This ethical discussion is, in fact, about the functioning of institutions and practices and what the providers see as ADL. The importance of arts and other cultural activities as ADL has long been acknowledged. The arts not only have a positive impact on users’ stress, mood, pain levels and sleep but also enhance communication between staff and users (Cann, 2017; Knudtsen, Holmen, & Håpnes, 2005; Solli, Rolvsjord, & Borg, 2013; Wilson, Bungay, Munn‐Giddingsa, & Boyce, 2016). Facilitating a climate where to achieve flourishing is necessary, but still a challenge. To shift to a flourishing perspective, to think outside the box and to act on that shift perspective, is thus a courageous, albeit demanding, step for the carers (Cann, 2017; Horghagen, 2016). It demands institutional policies that support these carers. Only then, the users’ wishes and values can be met in a respectful way.

The education program Active Care is the only known education programme that teaches carers how to implement the notion of active ageing through (cultural) activities of daily living. The aim—to offer users the opportunity to promote, restore and maintain health—is achieved by implementing new and creative activities in health care. This involves, among other things, assessment of the person's wishes and working together to decide which activities are appropriate. This reciprocity seems to spark further actions among those involved, either spontaneous or planned. This is in agreement with the description of human flourishing (Dewing & McCormack, 2017) and also confirms that experiences are related to contexts and relationships (Clandinin et al., 2016). This indicates that the Active Care education programme helped the carers to promote active ageing. Active ageing can thus be a means to promote flourishing.

Despite the White Papers reorientation to Active Care, (cultural) activities are often not part of ADL—even though it has been shown that users want more activities and that activities reduce overall costs (Conner, Young, & Silvia, 2016; Disch & Lorentzen, 2012; Haukvik & Eckhoff, 2017; Hofstad, 2018; Jensen, Stickley, Torrissen, & Stigmar, 2017; Johansen, Skaalvik Wollf, & Danielsen, 2018; Verhaeghe et al., 2014). An education programme such as Active Care appears is a way to help carers to become aware of the possibilities the cultural activities offer to achieve flourishing organizations. When flourishing is used as a measurable outcome in care, these outcomes can be lined up with institutional policies and contribute to the justness of the overall institution (Hewitt, 2017; Hone et al., 2014).

### 6.1 Implications

Because flourishing meets the WHO goal for well‐being despite disease and disability, carers need to be aware of the positive effects cultural activities have on their users, the team and workplace. However, it is important that the carers are offered an education programme to support such a change. Further, educational institutes need to incorporate activities as part of ADL in their curriculum. Finally, further research on the long‐term effects of cultural activities as ADL in care fields is needed.

### 6.2 Limitations

This study is based on data from students participating in an education programme. The findings, therefore, only reflect the participants experience at the end of the educational programme. Most students were not familiar with the concept of writing practice narratives. Therefore, the quality of these narratives differed a great deal. Many reflections, adjustments, decisions and struggles in the process are not described in the narratives. However, the material gave sufficient insight into the carers’ experiences.

## 7 CONCLUSIONS

This study shows that the Active Care education program contributes to more and new activities, to good relationships and to positive emotions. Cultural activities are a basic human right and ought to be seen as important as any other ADL. To achieve this, flourishing should be seen as an important outcome in health care.

The results of this study have indicated what promotes well‐being in a healthcare setting. With the demographic challenges in mind, it seems necessary to take steps to value cultural activities as part of ADL. The shift to more person‐ centred care reminds us of what is valuable and of what gives meaning for a person in his or her life. The Active Care education programme is valuable for such a shift.

## CONFLICTS OF INTEREST

None.

## AUTHOR CONTRIBUTIONS

JV designed the study, collected the data, analysed the data and mainly drafted the manuscript. SH contributed to the analysis, revised the manuscript critically for scientific content, read and approved the final version.
